# Behavioural insights and the evolving COVID-19 pandemic

**DOI:** 10.2807/1560-7917.ES.2022.27.18.2100615

**Published:** 2022-05-05

**Authors:** Marijn de Bruin, Jonathan E Suk, Marianna Baggio, Sarah Earnshaw Blomquist, María Falcon, Maria João Forjaz, Karina Godoy-Ramirez, Mariken Leurs, Carmen Rodriguez-Blazquez, María Romay-Barja, Ellen Uiters, John Kinsman

**Affiliations:** 1 National Institute for Public Health and the Environment (RIVM), Bilthoven, the Netherlands; 2 Radboud University Medical Center, Institute of Health Sciences, Nijmegen, the Netherlands; 3 European Centre for Disease Prevention and Control (ECDC), Solna, Sweden; 4 Joint Research Centre, European Commission, Brussels, Belgium; 5 The Public Health Agency of Sweden, Solna, Sweden; 6 Department of Legal and Forensic Medicine, Faculty of Medicine, Biomedical Research Institute (IMIB), University of Murcia, Murcia, Spain; 7 National Epidemiology Centre, Carlos III Health Institute, Madrid, Spain; 8 Research Network on Health Services in Chronic Diseases (REDISSEC), Madrid, Spain; 9 National Centre of Tropical Medicine, Carlos III Health Institute, Madrid, Spain; 10 Infectious Disease Network Research Center (CIBERINFEC), Madrid, Spain

**Keywords:** Behavioural science, prevention, pandemic, COVID-19, vaccination, pandemic preparedness

## Abstract

Behavioural sciences have complemented medical and epidemiological sciences in the response to the SARS-CoV-2 pandemic. As vaccination uptake continues to increase across the EU/EEA – including booster vaccinations – behavioural science research remains important for both pandemic policy, planning of services and communication. From a behavioural perspective, the following three areas are key as the pandemic progresses: (i) attaining and maintaining high levels of vaccination including booster doses across all groups in society, including socially vulnerable populations, (ii) informing sustainable pandemic policies and ensuring adherence to basic prevention measures to protect the most vulnerable population, and (iii) facilitating population preparedness and willingness to support and adhere to the reimposition of restrictions locally or regionally whenever outbreaks may occur. Based on mixed-methods research, expert consultations, and engagement with communities, behavioural data and interventions can thus be important to prevent and effectively respond to local or regional outbreaks, and to minimise socioeconomic and health disparities. In this Perspective, we briefly outline these topics from a European viewpoint, while recognising the importance of considering the specific context in individual countries.

## Background

Behavioural sciences – including cognitive psychology, anthropology, social psychology, behavioural economics, sociology and other areas – are an essential complement to medical and epidemiological sciences in the response to the coronavirus disease (COVID-19) pandemic [1]. European Union/European Economic Area (EU/EEA) countries are at different stages in their application of behavioural science in COVID-19 responses [2]. Studies have been conducted in many countries to monitor public acceptance of and adherence to non-pharmaceutical interventions (NPIs) aimed at restricting the spread of COVID-19, as well as their impact on broader public health issues such as mental health. There has also been extensive work aimed at gaining insights into acceptance and uptake of COVID-19 vaccination [3-5]. These insights have permeated to varying degrees into COVID-19 policies, the planning of services and public health communication strategies. However, despite the high levels of immunity in the population (be it from vaccination or through infection, anno May 2022), there still remain – from a behavioural perspective – multiple important challenges and uncertainties. The latter include the population willingness to repeatedly receive booster vaccinations, seasonal effects on severe acute respiratory syndrome coronavirus-2 (SARS-CoV-2) transmission and its associated morbidity, and the potential rise of variants of concern (VOC), which could lead to the reimposition of prevention measures and further test countries’ resilience in dealing with COVID-19.

As a group of behavioural scientists working in/with national and international public health institutes across Europe, we present here a Perspective that highlights several ongoing challenges that behavioural science research could help to address: (i) attaining and maintaining high levels of (booster) vaccination coverage across all adults, adolescents and children, especially in socially and medically vulnerable populations; (ii) informing sustainable pandemic policies and supporting continued adherence to recommended prevention measures to protect vulnerable people; and (iii) facilitating population preparedness and willingness to support and adhere to the local or regional reimposition of restrictions in the event of new outbreaks with high morbidity and/or mortality rates.

The impact of vaccination in reducing transmission of SARS-CoV-2, but especially severe outcomes such as hospitalisations and mortality, has by now been well-established [6,7]. However, previous ‘waves’ of the pandemic have been driven – on the one hand – by new and more transmissible VOC, but on the other, by the relaxation of COVID-19 prevention measures, international travels [8] and increased social contacts. Given the potential for such processes to continue to facilitate future waves of SARS-CoV-2 Omicron (Phylogenetic Assignment of Named Global Outbreak (Pango) lineage designation B. 1.1.529) and/or other emerging VOC, it is important to continue to promote prevention measures in order to protect the most vulnerable and prepare for dealing with local or more widespread outbreaks [9].

From a behavioural perspective, these issues are an important part of the strategic considerations that can impact on the implementation of recommendations. Until recently, people have been living under restrictive NPIs for almost 2 years, the negative impacts of which have been substantial, such as increases in poor mental health coinciding with economic and educational losses [10]. Were there to be another upsurge in cases, authorities may face population and stakeholder resistance to the continuation or reimposition of NPIs, especially those that substantially affect people’s social life or involve closures of venues such as educational institutions, shops or restaurants [11].

## Behavioural challenges and uncertainties

In broad terms, to protect the most vulnerable people, two elements may be needed: the prevention of transmission through sustained, targeted NPIs and successful (booster) vaccination programmes. In addition, it is important to develop a longer-term plan for sustainable control of the virus while keeping societies as open as possible, but also to prepare for possible setbacks by having a well-defined plan –broadly supported by citizens and societal stakeholders –for how to deal with new outbreaks (seasonal or VOCs). Each of these challenges will be briefly discussed below from a behavioural science perspective.

### Vaccination uptake

The many factors that can influence whether or not a population ends up being well-protected by COVID-19 vaccines can be categorised into supply-side issues and demand-side issues. From a social and behavioural perspective, we are mainly focussed on gaining insights into the demand side issues: what people think and feel about the vaccines, including perceptions about vaccine safety and effectiveness; social processes such as trust in authorities and social norms in communities and workplaces; motivation to vaccinate, e.g. with the incentive to protect oneself or others against serious disease or to return to a more normal life; and practical barriers that make it more or less easy to access vaccination [12]. Each of these issues can differ between groups in the population, and within people over time as more information about vaccine safety and effectiveness becomes available.

From a behavioural perspective, it is essential to monitor COVID-19 vaccination intentions and uptake over time (following first, second and booster doses), and also specifically in groups of the population which may be less likely to participate in government surveys. Both quantitative and qualitative research is required to understand people’s perceptions, intentions and behaviour in order to inform communication activities, i.e. what information should be communicated, by whom, how and for how long. In order to reduce socioeconomic disparities, a priority is to engage with groups in the population with lower levels of trust in the government and those who do not generally access traditional media, who may be both less likely to participate in this research and to have lower vaccine uptake [13,14].

Behavioural research from previous vaccination campaigns and the current COVID-19 vaccination programme suggests that campaigns to promote and support vaccine uptake should at least focus on these three steps: (i) information – transparently informing people about the risks and benefits of the vaccines, including how they were developed; (ii) motivation – linking vaccination to personal and collective values and norms, such as the desire to protect one’s own health, to protect others, and to speed up relaxation of restrictive measures; and (iii) behaviour – making vaccination locations easily accessible and using cues and prompts to remind people of their appointments [3].

It is key that information provision is done by people or organisations who are perceived by the information recipient as trustworthy experts, and that recipients have the opportunity to discuss concerns and ask questions. Whereas for some people mass media messages and online communication suffices, other people may prefer contact with their general practitioner (GP), pharmacist or a public health professional in their own neighbourhood [1,15,16]. As this could require additional time from healthcare professionals already under considerable pressure, complementary modalities such as a national ‘vaccine hesitation telephone line’ run by trained medical students, e.g. https://twijfeltelefoon.nl in the Netherlands, could be considered. Behavioural research can provide further guidance in all these areas, thereby ensuring relevance and acceptability of the different vaccination strategies and messages to the different target populations. Since the vaccination programme for COVID-19 is likely to be needed for some time to come, this research should continue to facilitate its long-term success.

### Maintaining adherence to basic prevention measures 

The phenomenon of ‘pandemic fatigue’ has been defined by WHO as ‘distress which can result in demotivation to follow recommended protective measures’ [17]. It is important to examine the source of this demotivation [18] and recognise that it may not apply equally to all measures. Firstly, studies and ongoing national surveys suggest that people experience the most negative impact from measures that limit social participation. Hygiene behaviours, such as regularly washing hands, face masks or testing for COVID-19 when having symptoms, seem to be easier to maintain over time. Secondly, a reduction in adherence to basic prevention measures following the SARS-CoV-2 Omicron VOC surge across Europe will at least in part be due to reduced perceptions of risk and usefulness of pandemic restrictions, given the population immunity and reductions in hospital admissions. Moreover, also the consequent relaxation of restrictions affects people’s motivation (lower perceived necessity) and their opportunity to follow recommendations, as for example physical distancing is much more difficult in crowded places [19]. From a behavioural perspective, it thus seems a priority to inform people which NPIs are required under which particular epidemiological and social contexts and why, with a focus on protecting people who are medically vulnerable. It is also important that the prevention measures being maintained are both sufficiently effective (epidemiological evidence) and feasible (behavioural evidence) for people to follow through the full implementation timeframe. Behavioural expertise is required to help identify those NPIs, and to develop the message, support and conditions to facilitate behavioural maintenance.

### Contingency plans

Vaccination rates have slowed down in many countries, and although repeat booster campaigns are underway to maintain high levels of population immunity, coverage is suboptimal in some groups and regions – and it is unsure how many vaccinated people will decide to continue taking booster shots. This implies that there are some groups remaining in the population who continue to face barriers to vaccination acceptance and uptake. While the immunity in the population for SARS-CoV-2 Omicron VOC substantially reduces disease severity, other VOCs could partially evade the protection against severe disease offered by vaccination. If these occur alongside increased mobility and social mixing and/or a general reduction in adherence to NPIs, then local or regional outbreaks that require at least temporary reimposition of restrictive NPIs seem plausible. 

In case of the reimposition of NPIs, national, regional or local authorities and communication teams will then need to manage the population’s uncertainty, disappointment and possibly resistance that could accompany such a scenario. For example, research among the general public and societal stakeholders in the Netherlands shows relatively high support (> 70%) for maintaining low-impact measures such as washing hands, (self-)testing and ventilation even in an endemic scenario; however, when confronted with a VOC scenario in which the healthcare system comes under severe pressure, a minority of people would opt for more stringent measures such as closing shops, restaurants, a curfew or cancelling large events [11]. From a behavioural perspective, it is therefore paramount to proactively develop contingency plans – together with representatives from key community stakeholders, e.g. local municipalities, police, shop owners, educational institutions, citizens, religious leaders – in order to create realistic strategies with a broad basis of support. Communicating about possible scenarios and contingency plans early may make it more likely that when they need to be put into practice, this will be done effectively and without undue resistance.

## Conclusions

We have outlined three important behavioural challenges that continue to be relevant in this COVID-19 pandemic. These challenges concern ensuring high vaccination uptake in all eligible population groups, maintaining adherence to selected NPIs, and creating and effectively implementing a contingency ‘plan B’ with strong support from societal stakeholders and citizens in order to respond effectively to any local or regional resurgence of the virus. We have focused on briefly outlining these challenges, the behavioural complexities involved and identified indicative directions for managing these (Box).

Box **Examples of how behavioural insights can inform long-term pandemic policies**

- Contributing to policies: selection of effective, acceptable, and feasible measures for longer-term prevention as well as for contingency planning.
- Modifying the physical context: facilitating behaviours by organising vaccination locally or ensuring accessibility to (free) lateral flow tests (COVID-19 self-tests); or creating safer schools and workplaces through supporting the implementation of prevention measures such as adequate ventilation.
- Informing services and facilities: identifying services to support adherence and well-being, such as free COVID-19 testing or supported isolation.
- Effective communication: proactive, consistent, reliable and transparent communication (as perceived by recipients) addressing population needs and perceptions, including methods and techniques for behavioural change and maintenance.
- Stakeholder engagement: effectively involving citizens and societal stakeholders to develop long-term pandemic management plans that are feasible and broadly supported

Scientific evidence that behavioural strategies – such as risk communication or modifying the physical environment – are effective in the context of the COVID-19 pandemic has been steadily accumulating [20-23], although much of the available evidence has not yet been published in scientific journals. Putting these insights into practice will be a continued multi-disciplinary effort that incorporates research and expert consensus methods from behavioural and medical sciences, as well as engagement with stakeholders and citizens, to collaboratively deal with the uncertainties that lie ahead – uncertainties both in terms of the evolution of the virus as well as the social and behavioural processes that will be needed to optimise the long-term success of COVID-19 prevention policies.

## References

[1] Bavel JJV Baicker K Boggio PS Capraro V Cichocka A Cikara M Using social and behavioural science to support COVID-19 pandemic response. Nat Hum Behav. 2020;4(5):460-71. 10.1038/s41562-020-0884-z 32355299

[2] European Centre for Disease Prevention and Control (ECDC). Behavioural Insights research to support the response to COVID 19: a survey of implementation in the EU/EEA. Stockholm: ECDC; 17 Feb 2021. Available from: https://www.ecdc.europa.eu/en/publications-data/behavioural-insights-research-support-response-covid-19

[3] Sanders JG Spruijt P van Dijk M Elberse J Lambooij MS Kroese FM Understanding a national increase in COVID-19 vaccination intention, the Netherlands, November 2020-March 2021. Euro Surveill. 2021;26(36):2100792. 10.2807/1560-7917.ES.2021.26.36.2100792 34505565 PMC8431991

[4] World Health Organization (WHO). Data for action: achieving high uptake of COVID-19 vaccines. Geneva: WHO; 2021. Available from: https://www.who.int/publications/i/item/WHO-2019-nCoV-vaccination-demand-planning-2021.1

[5] Folkhälsomyndigheten. Resultat för befolkningens acceptans för vaccination mot covid-19. [Results for population acceptance for vaccination against covid-19]. Stockholm: Folkhälsomyndigheten; 2021. Swedish. Available from: https://www.folkhalsomyndigheten.se/smittskydd-beredskap/utbrott/aktuella-utbrott/covid-19/statistik-och-analyser/acceptans-for-vaccination-mot-covid-19

[6] Christie A Henley SJ Mattocks L Fernando R Lansky A Ahmad FB Decreases in COVID-19 cases, emergency department visits, hospital admissions, and deaths among older adults following the introduction of COVID-19 vaccine — United States, September 6, 2020–May 1, 2021. MMWR Morb Mortal Wkly Rep. 2021;70(23):858-64. 10.15585/mmwr.mm7023e2 34111059 PMC8191865

[7] Chemaitelly H Ayoub HH AlMukdad S Coyle P Tang P Yassine HM Duration of mRNA vaccine protection against SARS-CoV-2 Omicron BA.1 and BA.2 subvariants in Qatar. medRxiv 2022.03.13.22272308. Preprint. 10.1101/2022.03.13.22272308 PMC9163167 35654888

[8] Lemey P Ruktanonchai N Hong SL Colizza V Poletto C Van den Broeck F Untangling introductions and persistence in COVID-19 resurgence in Europe. Nature. 2021;595(7869):713-7. 10.1038/s41586-021-03754-2 34192736 PMC8324533

[9] European Centre for Disease Prevention and Control (ECDC). Rapid risk assessment: Assessing SARS-CoV-2 circulation, variants of concern, non-pharmaceutical interventions and vaccine rollout in the EU/EEA, 15th update. Stockholm: ECDC; 10 Jun 2021. Available from: https://www.ecdc.europa.eu/en/publications-data/rapid-risk-assessment-sars-cov-2-circulation-variants-concern

[10] Xiong J Lipsitz O Nasri F Lui LMW Gill H Phan L Impact of COVID-19 pandemic on mental health in the general population: A systematic review. J Affect Disord. 2020;277:55-64. 10.1016/j.jad.2020.08.001 32799105 PMC7413844

[11] Uiters E, Kroese F, Spruijt P, Kolner C, Wuyts R, Geijsen T, et al. De langetermijnaanpak van het coronabeleid: voorkeur van burgers en het maatschappelijk middenveld. [The long-term corona strategy: preferences from citizens and societal stakeholders]. Bilthoven: Rijksinstituut voor Volksgezondheid en Milieu; 2022. Dutch. Available from: https://www.rivm.nl/documenten/langetermijnaanpak-van-coronabeleid-voorkeur-van-burgers-en-maatschappelijk-middenveld

[12] Brewer NT Chapman GB Rothman AJ Leask J Kempe A . Increasing vaccination: putting psychological science into action. Psychol Sci Public Interest. 2017;18(3):149-207. 10.1177/1529100618760521 29611455

[13] Robinson E Jones A Lesser I Daly M . International estimates of intended uptake and refusal of COVID-19 vaccines: A rapid systematic review and meta-analysis of large nationally representative samples. Vaccine. 2021;39(15):2024-34. 10.1016/j.vaccine.2021.02.005 33722411 PMC7867398

[14] Fransen M, Uiters E. Vaccinatiebereidheid COVID-19 onder groepen met een migratieachtergrond. [Vaccination readiness for COVID-19 among groups with a migration background]. Bilthoven: Rijksinstituut voor Volksgezondheid en Milieu; 2021. Dutch. Available from: https://www.rivm.nl/documenten/vaccinatiebereidheid-covid-19-onder-groepen-met-migratieachtergrond

[15] Bach AT Kang AY Lewis J Xavioer S Portillo I Goad JA . Addressing common barriers in adult immunizations: a review of interventions. Expert Rev Vaccines. 2019;18(11):1167-85. 10.1080/14760584.2019.1698955 31791159

[16] Butler R MacDonald NE SAGE Working Group on Vaccine Hesitancy . Diagnosing the determinants of vaccine hesitancy in specific subgroups: The Guide to Tailoring Immunization Programmes (TIP). Vaccine. 2015;33(34):4176-9. 10.1016/j.vaccine.2015.04.038 25896376

[17] World Health Organization Regional Office for Europe (WHO/Europe). Pandemic fatigue: reinvigorating the public to prevent COVID-19: policy considerations for Member States in the WHO European Region. WHO/Europe; 2020. Available from: https://apps.who.int/iris/handle/10665/335820

[18] Michie S West R Harvey N . The concept of "fatigue" in tackling covid-19. BMJ. 2020;371:m4171. 10.1136/bmj.m4171 33139254

[19] Liebst L Ejbye-Ernst P de Bruin M Thomas J Lindegaard M . Mask-wearing and social distancing: Evidence from a video-observational and natural-experimental study of public space behavior during the COVID-19 pandemic. PsyArXiv. 2021. Preprint. 10.31234/osf.io/ep8jg

[20] European Centre for Disease Prevention and Control (ECDC). SARS-CoV-2 variants of concern as of 1 July 2021. Stockholm: ECDC. [Accessed: 6 Jul 2021]. Available from: https://www.ecdc.europa.eu/en/covid-19/variants-concern

[21] Haug N Geyrhofer L Londei A Dervic E Desvars-Larrive A Loreto V Ranking the effectiveness of worldwide COVID-19 government interventions. Nat Hum Behav. 2020;4(12):1303-12. 10.1038/s41562-020-01009-0 33199859

[22] Dai H Saccardo S Han MA Roh L Raja N Vangala S Behavioural nudges increase COVID-19 vaccinations. Nature. 2021;597(7876):404-9. 10.1038/s41586-021-03843-2 34340242 PMC8443442

[23] Abaluck J Kwong LH Styczynski A Haque A Kabir MA Bates-Jefferys E Impact of community masking on COVID-19: A cluster-randomized trial in Bangladesh. Science. 2022;375(6577):eabi9069. 10.1126/science.abi9069 34855513 PMC9036942

